# Marital Status and Gender Differences as Key Determinants of COVID-19 Impact on Wellbeing, Job Satisfaction and Resilience in Health Care Workers and Staff Working in Academia in the UK During the First Wave of the Pandemic

**DOI:** 10.3389/fpubh.2022.928107

**Published:** 2022-06-27

**Authors:** Junjie Peng, Wing Han Wu, Georgia Doolan, Naila Choudhury, Puja Mehta, Ayesha Khatun, Laura Hennelly, Julian Henty, Elizabeth C. Jury, Lih-Mei Liao, Coziana Ciurtin

**Affiliations:** ^1^Division of Medicine, Centre for Adolescent Rheumatology Research, University College London, London, United Kingdom; ^2^Medical School, University College London, London, United Kingdom; ^3^Department of Medicine, Centre for Inflammation and Tissue Repair, University College London Respiratory, University College London, London, United Kingdom; ^4^Department of Clinical and Health Psychology, St Mary's Hospital, London, United Kingdom; ^5^Division of Medicine, Centre for Rheumatology Research, University College London, London, United Kingdom; ^6^Women's Health Psychological Services, University College London, London, United Kingdom

**Keywords:** wellbeing, job satisfaction, resilience, health care workers, COVID-19 pandemic in the UK

## Abstract

**Background:**

The COVID-19 pandemic is an unprecedented global public health crisis that continues to exert immense pressure on healthcare and related professional staff and services. The impact on staff wellbeing is likely to be influenced by a combination of modifiable and non-modifiable factors.

**Objectives:**

The aim of this study is to evaluate the effect of the COVID-19 pandemic on the self-reported wellbeing, resilience, and job satisfaction of National Health Service (NHS) and university staff working in the field of healthcare and medical research.

**Methods:**

We conducted a cross sectional survey of NHS and UK university staff throughout the COVID-19 pandemic between May-November 2020. The anonymous and voluntary survey was disseminated through social media platforms, and via e-mail to members of professional and medical bodies. The data was analyzed using descriptive and regression (R) statistics.

**Results:**

The enjoyment of work and satisfaction outside of work was significantly negatively impacted by the COVID-19 pandemic for all of staff groups independent of other variables. Furthermore, married women reporting significantly lower wellbeing than married men (*P* = 0.028). Additionally, the wellbeing of single females was significantly lower than both married women and men (*P* = 0.017 and *P* < 0.0001, respectively). Gender differences were also found in satisfaction outside of work, with women reporting higher satisfaction than men before the COVID-19 pandemic (*P* = 0.0002).

**Conclusion:**

Our study confirms that the enjoyment of work and general satisfaction of staff members has been significantly affected by the first wave of the COVID-19 pandemic. Interestingly, being married appears to be a protective factor for wellbeing and resilience but the effect may be reversed for life satisfaction outside work. Our survey highlights the critical need for further research to examine gender differences using a wider range of methods.

## Introduction

In December 2019, The Wuhan Municipal Health Commission reported a cluster of cases of an atypical pneumonia in Wuhan, China, which was later attributed to a novel coronavirus termed ‘severe acute respiratory syndrome coronavirus 2' (SARS-CoV-2)12. The COVID-19 pandemic was declared by the World Health Organization (WHO) on the 11th March 2020 and, as of November 2021, there have been over 258 million cases and 5.18 million deaths worldwide, with more than 9 million cases and 144,000 deaths reported in the UK (1).

In the UK, the mental health effects on the general population have attracted significant research interests. It was suggested that the prevalence of depression had increased from 10% before the pandemic (July 2019–March 2020) to 21% during the UK's second wave of the pandemic (January 2021–March 2021). These findings, reported by the Office for National Statistics (ONS), also identified additional risk factors for depression, including female gender, age 16–39 years old, the presence of a disability, unemployment, living in a deprived area and the inability to afford an unexpected expense (2).

In general, health care workers (HCWs) are known to report higher levels of depression, anxiety, and stress compared to the general population (3), particularly affecting nurses and female staff in general (4). Unsurprisingly, recent research has shown that the COVID-19 pandemic has affected health professionals across the world (5–13). Risk factors associated with poorer psychological wellbeing in HCWs throughout the pandemic included age, sex and marital status. Being younger (9, 14–18) as well as older (19) correlated with poorer outcomes, while almost consistently, being a female had a negative impact of mental health during the pandemic (6–8, 11, 16–18, 20). Being single was more commonly associated with negative outcomes (19, 21, 22); however, one study focused on HCWs from the Eastern Mediterranean region reported alternative findings that being married was associated with reduced psychological wellbeing (23).

A study in Finland observed heightened levels of anxiety amongst all surveyed hospital workers, but this was found to be independent to their exposure to COVID-19 cases (14). Other studies found differences in wellbeing between occupational groups. Several studies have identified nurses to be the profession most at risk (7, 8, 13, 16, 17, 24, 25), while only a few studies have found physicians to have a higher level of stress (23) and depression (26) than other HCWs during the COVID-19 pandemic. Numerous studies have found an association between working on the frontline and lower psychological wellbeing (5, 7, 9, 10, 18, 27). A large US based study of 5,550 clinical and non-clinical staff reported that anxiety, depression, and high levels of work exhaustion were independently associated with community or clinical exposure to COVID-19 (28). However, two studies have found that HCWs working on the frontline actually reported better psychological wellbeing compared to non-frontline staff (29, 30). The researchers postulated that this may be due to a greater sense of control and awareness of the situation. Another study from Singapore found that non-medical HCWs reported more anxiety compared to medical HCWs (31). Of interest is a study from Ethiopia that found that HCWs who perceived themselves as being at risk if infected with COVID-19 were four times more likely to be depressed in comparison to their colleagues (32), which points to the relevance of various psychological variables and personal views related to the individual risk of COVID-19 infection.

In contrast with negative outcomes, many studies investigated the resilience of HCWs, which is defined as the ability to positively adapt to traumatic or adverse experiences (33). As expected, the stress associated with life-style changes and pressures at work in the context of COVID-19 pandemic manifested in different coping behaviors with impact on the quality of life of HCWs. An integrative review explored the direct association between resilience and work engagement and social support, as well as negative correlations with anxiety and depression (34). In addition, some studies also highlighted geographic differences between the US (35) and China (36), where the pandemic was associated with a decrease vs. an increase in nurses' resilience levels compared to pre-pandemic levels.

Research appears to have yielded contradictory findings in terms of which were the most vulnerable HCW groups and what type of support is likely to be required to mitigate for the consequences of the COVID-19 pandemic on the mental health and quality of life of HCWs. Many discrepancies highlighted by the literature are potentially explained by the large number of variables involved, including dictinct regional conditions, clinical environment, changes to work patterns and the amount of perceived control and risks while at work, aspects that vary significantly between occupational groups and within the hierarchy of each professional group.

The aim of our study was to examine the effect of the COVID-19 pandemic on the mental health and wellbeing of National Health System (NHS) and University staff working in the field of healthcare and medical research in the UK. This was an exploratory survey focused on self-reported levels of wellbeing, resilience, and job satisfaction of staff both before (reported retrospectively) and during the COVID-19 pandemic (reported in real-time). Our hypothesis was that the COVID-19 pandemic significantly affected the outcomes described above.

In addition to investigating the hypothesis above, we aimed at identifying and investigating the impact of various individual variables (as detailed below) on the mental health and wellbeing of both NHS and university staff during the first wave of COVID-19 pandemic in the UK. The intention was to guide the development of targeted support measures for staff, with a particular focus on staff members who have been highlighted in research as being potentially more vulnerable.

## Methods

### Survey Design

We conducted a cross-sectional survey using Microsoft Forms (online platform) targeting NHS and university staff working in the UK through the COVID-19 pandemic between May and November 2020. The survey was disseminated through various social media platforms as well as being distributed to members of professional and medical bodies *via* e-mail.

Although our survey did not cover the whole period of COVID-19 pandemic in the UK, we took into consideration the timing of the government-imposed lockdowns and their potential influence on our collected outcomes. From 16th March 2020, the UK population was advised to avoid all non-essential traveling. Lock-down measures came into force on 26th March 2020 and were lifted nationally on 23rd of June. Further local lockdowns were imposed on the 4th July 2020. On the 14th August 2020 local restrictions were eased up to 14th October 2020 when a new three-tier system of restrictions in England.

The inclusion criteria for this study were as follows: (i) participants aged 18 years and above; and (ii) individuals who self-identified as working in a field related to healthcare; and (iii) ability to read and interpret the English language.

Approval was gained from relevant ethical bodies (UK Health Research Authority approval ref. IRAS ID 284105). Participation was both anonymous and voluntary, with implied consent. All participants were permitted to withdraw from the survey at any time by not completing or submitting their results.

## Questionnaire

Our survey consisted of 36 questions which gathered information on socio-demographic status, professional responsibilities, personal exposure to covid-19, remote working and redeployment, alongside self-reported levels of satisfaction, wellbeing, and resilience. The Content of the survey was analyzed and approved by an expert body that included academics, psychologists and regulatory bodies (UK Health Research Authority approval, reference: 20/HRA/2547). The respondents did not receive any incentive to complete the survey.

We collected data on various participant characteristics (predictors).

### Socio-Demographic Information

Participants were asked questions on their age, gender, ethnicity, marital status, education level, and area of residence.

### Professional Role and Responsibilities

Participants responded to various questions relating to their professional role and responsibilities including, job title, level of training and expertise, and area of work (community, research, pharmacy, or hospital setting).

### Exposure to COVID-19

Individuals were questioned on their exposure to COVID-19, including personal illness with COVID-19, isolation during the pandemic, and direct exposure to COVID-19 positive cases through work or personal contacts.

### Remote Working

Individuals were asked questions on their exposure to remote working, including changes to work environment because of the COVID-19.

### Redeployment

Individuals were question on whether they had been redeployed during the COVID-19 pandemic. Individuals were asked to report their levels of anxiety related to redeployment on a visual analog scale (VAS) from 1 to 10.

We also collected data on psychological outcomes, such as:

### Wellbeing

The Warwick-Edinburgh Mental Wellbeing Scale (WEMWBS) (37) is a validated tool with high internal validity for general population. The scale consisted of 14 items detailing statements about positive feelings and thoughts (rated from 1 - “none of the time” to 5 - “all the time”). The highest the value the higher the wellbeing. The value for Cronbach's Alpha for our survey was 0.94 (see Appendix 1 for the details related to the wellbeing aspects assessed by this validated scale).

### Resilience

The Resilience Scale (38) was initially developed to evaluate the levels of resilience in the general population. The shorter version is a 14-item scale, which is an abbreviated and validated version of the initial Resilience Scale (39). Higher scores indicate higher levels of resilience. The Cronbach's Alpha for our survey was 0.81 (see Appendix 2 for the details of the resilience aspects assessed by this scale).

### Satisfaction

The satisfaction of staff was assessed by asking individuals to score and compare their perceived levels (VAS scale 1–10) of job enjoyment and satisfaction outside of work from both before and during the COVID-19 pandemic using a single item approach (40). Higher scores indicate higher levels of satisfaction. The Cronbach's Alpha for our survey was 0.83.

## Statistical Analysis

### Sample Calculation

There are ~1.5 million of NHS and 0.5 million Higher Education employees in the UK. According to UK Government statistics approximately one in seven NHS workers have been redeployed during the first wave of the pandemic (Have NHS staff been redeployed due to COVID-19? | YouGov). For a 10% margin of error in the estimation of wellbeing, resilience and job satisfaction of a total population of two million relevant professionals (even if not everyone was directly impacted through their work by the COVID-19 pandemic), the required sample size was 97, while for a 5% margin of error, the sample size required was 384.

### Analysis

The data were collated using an Excel spreadsheet and analyzed using descriptive statistics based on the type of data distribution (normality test). We used R package (4.2.0) for statistical analysis: Student's *t-*test (for comparisons between two groups of variables with normal distribution) and Mann-Whitney U Test (for variables with skewed distribution), Fisher's exact test to assess for associations between two categorical variables, Welch's *t*-test (to test the hypothesis that two populations have equal means which we applied when comparing the male and female staff groups). We used linear regression to predict the outcome of interest (resilience, wellbeing or job satisfaction) when accounting for independent variables (such as gender, marital status, and other factors described above under *predictors*). *P* < 0.05 was considered statistically significant.

## Results

### Characteristics of Survey Respondents

A total of 365 responses were received during the period of 6 months (May-November 2020) when the survey link was active which was an adequate sample size for a low margin error (5%). As the survey was also disseminated via social media, we could not calculate a response rate. The average time for survey completion for the study participants was 9 min.

Age, gender, ethnicity, marital status, job roles, area of residence and proportion of respondents redeployed to patient facing roles during the pandemic are presented in Table 1 according to their status (single vs. in partnership vs. married). There were no significant differences between the three responder categories.

**Table 1:** Responders' characteristics presented according to their status (single vs. in partnership vs. married) which was identified as a key determinant of COVID-19 pandemic impact).

**Respondents' characteristics**	**Married**	**Single**	**Partnership**	***P*-values**
Number	197	94	74	-
**Age**				
• 18–25 • 26–30 • 31–40 • 41–50 • 51–60 • Over 60	• 0 • 4 • 56 • 70 • 48 • 19	• 8 • 19 • 32 • 18 • 15 • 2	• 2 • 20 • 27 • 10 • 11 • 4	
**Age (mean)**	47.10152	38.63298	39.28378	
**Gender**				Married vs. single:
• Female • Male • Other	• 118 • 79 • 0	• 74 • 19 • 1	• 55 • 19 • 0	*P* = 0.0005 Married vs. partnership: *P* = 0.03 Single vs. partnership: *P* = 0.57
**Ethnicity** • White • Non-white	• 132 • 65	• 60 • 34	• 63 • 11	Married vs. single: *P* = 0.60 Married vs. partnership: *P* = 0.004 Single vs. partnership: *P* = 0.002
**Area of residence** • Urban • Rural	• 170 • 27	• 87 • 7	• 66 • 8	Married vs. single: *P* = 0.17 Married vs. partnership: *P* = 0.68 Single vs. partnership: *P* = 0.59
**In a patient facing role** • Yes • No	• 159 • 38	• 73 • 21	• 51 • 23	Married vs. single: *P* = 0.54 Married vs. partnership: *P* = 0.05 Single vs. partnership: *P* = 0.22
**Redeployment to a patient facing role during COVID-19 pandemic**				Married vs. single:
• Yes • No	• 53 • 144	• 30 • 64	• 22 • 52	*P* = 0.41 Married vs. partnership: *P* = 0.65 Single vs. partnership: *P* = 0.87

### Impact of Respondents' Status (Single vs. in Partnership vs. Married) on Survey Outcomes

*Self-reported job-satisfaction and satisfaction outside work prior (retrospective reporting) and during the COVID-19 pandemic (current reporting)*.

We explored the impact of respondents' status (single vs. in partnership vs. married) on job-satisfaction and satisfaction outside work pre and during COVID-19 pandemic (Table 2). Job enjoyment was perceived as higher pre COVID as opposed to during the first wave of the pandemic in the UK in all three status groups.

**Table 2:** Self-reported job-enjoyment and satisfaction outside work prior (retrospective reporting) and during the COVID-19 pandemic (real-life reporting) are presented according to the responders' status (single vs. in partnership vs. married).

	**Married**	**Single**	**In partnership**	
**Job enjoyment** **prior to COVID-19 pandemic** (VAS 1–10) Mean (IQR)	7.589 (7.000–8.000)	7.021 (6.000–8.000)	7.243 (6.250–8.000)	Married vs. single: ***P*** **=** **0.003** Married vs. partnership: *P* = 0.45 Single vs. partnership: *P* = 0.15
**Job enjoyment** **during COVID-19 pandemic** (VAS 1–10) Mean (IQR)	5.513 (4.000–7.000)	5.351 (3.250–7.000)	5.514 (4.000–7.000)	Married vs. single: *P* = 0.54 Married vs. partnership: *P* = 0.78 Single vs. partnership: *P* = 0.57
**Job enjoyment** **difference prior vs. during COVID-19 pandemic** Mean (IQR)	2.076 (0.000–4.000)	1.670 (0.00–4.00)	1.730 (0.00–3.75)	Married vs. single: *P* = 0.37 Married vs. partnership: *P* = 0.32 Single vs. partnership: *P* = 0.92
**Job enjoyment** **prior vs during COVID-19 pandemic**	***P*** **<** **0.0001**	***P*** **<** **0.0001**	***P*** **<** **0.0001**	
**Satisfaction outside** **work prior COVID-19 pandemic** (VAS 1–10) Mean (IQR)	8.036 (7.000–9.000)	7.628 (7.000–9.000)	8.203 (8.000–9.000)	Married vs. single: ***P*** **=** **0.04** Married vs. partnership: *P* = 0.62 Single vs. partnership: ***P*** **=** **0.03**
**Satisfaction outside** **work during COVID-19 pandemic** (VAS 1–10) Mean (IQR)	5.477 (4.000–7.000)	4.723 (3.000–7.000)	5.703 (4.000–7.000)	Married vs. single: ***P*** **=** **0.02** Married vs. partnership: *P* = 0.47 Single vs. partnership: ***P*** **=** **0.01**
**Satisfaction outside** **work difference prior vs during COVID-19 pandemic** Mean (IQR)	2.558 (0.000–4.000)	2.904 (1.000–5.000)	2.500 (1.000–4.000)	Married vs. single: *P* = 0.28 Married vs. partnership: *P* = 0.86 Single vs. partnership: *P* = 0.28
**Satisfaction outside** **work prior vs. during COVID-19 pandemic**	***P*** **<** **0.0001**	***P*** **<** **0.0001**	***P*** **<** **0.0001**	

*Bold values indicate P < 0.05*.

Individuals in each marital group recalled significantly higher levels of job enjoyment before the COVID-19 pandemic when compared to during the pandemic, irrespective of their marital status (*P* < 0.0001). No significant difference was found between each marital group at the same time point.

Married staff reported higher levels of job enjoyment than those who are single before the COVID-19 pandemic (*P* = 0.003). Regarding job enjoyment of staff before the COVID-19 pandemic, no significant difference was observed between married staff and staff in partnerships (*P* = 0.45), or between single staff and those in partnerships (*P* = 0.15). There was no observed difference between the marital groups in job enjoyment during the COVID-19 pandemic.

*Self-reported wellbeing, resilience and anxiety related to redeployment during the COVID-19 pandemic (current reporting)*.

We evaluated the impact of responders' status (single vs. in partnership vs. married) on wellbeing, resilience and anxiety related to redeployment during the COVID-19 pandemic (Table 3).

**Table 3:** Self-reported wellbeing, resilience and anxiety related to redeployment during the COVID-19 pandemic (real-life reporting) are presented according to the responders' status (married vs. single vs. in partnership).

	**Married**	**Single**	**Partnership**	
**Redeployment-related anxiety during COVID-19 pandemic** (VAS 1–10) Mean (IQR)	7.268 (6.000–8.000)	6.684 (5.000–8.000)	7.059 (6.000–8.000)	Married vs. single: *P* = 0.36 Married vs. partnership: *P* = 0.55 Partnership vs. single: *P* = 0.74
**Wellbeing during COVID-19 pandemic** (VAS 1–5) Mean (IQR)	3.357 (2.923–3.769)	3.097 (2.692–3.615)	3.180 (2.788–3.596)	Married vs. single: ***P*** **=** **0.002** Married vs. partnership: ***P*** **=** **0.04** Partnership vs. single: *P* = 0.42
**Resilience during COVID-19 pandemic** (VAS 1–7) Mean (IQR)	5.416 (4.714–6.071)	4.960 (4.304–5.643)	5.186 (4.643–5.786)	Married vs. single: ***P*** **=** **0.0006** Married vs. partnership: ***P*** **=** **0.04** Partnership vs. single: *P* = 0.25

*Bold values indicate P < 0.05*.

Married staff overall perceived their wellbeing as significantly higher than single members of staff and those in partnerships (*P* = 0.002, *P* = 0.04, respectively). There was no significant difference in the wellbeing of single staff vs. those in partnerships either (*P* = 0.42).

The perceived resilience of married staff was significantly higher than their single counterparts (*P* = 0.0006) or staff currently in partnership (*P* = 0.04). No significant difference was observed in the resilience between married staff and those who were single (*P* = 0.25).

### Impact of Responders' Gender and Marital Status on Survey Outcomes

**Figure 1:**
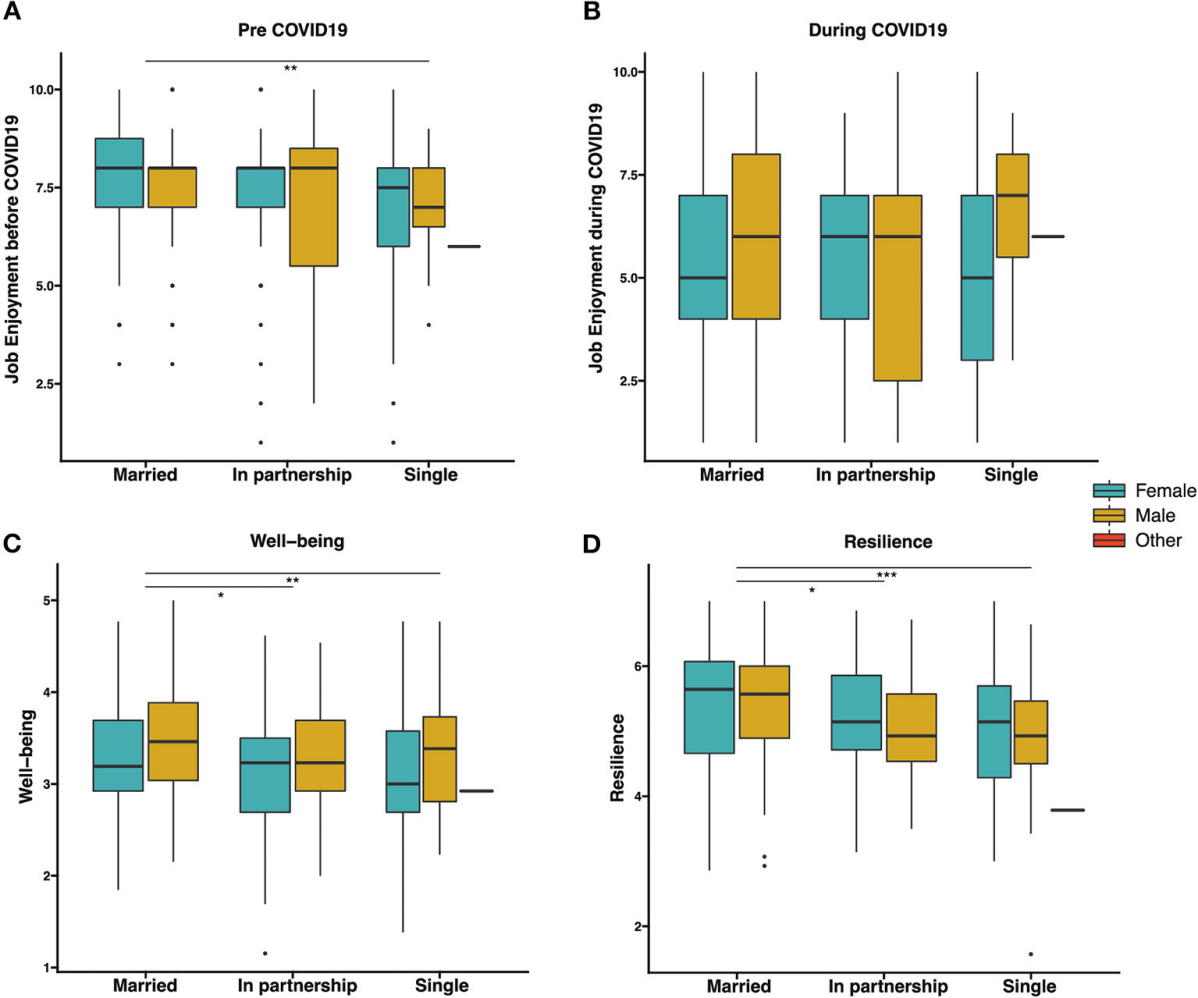
Box plots comparing job enjoyment (before COVID-19 or during COVID-19), well-being and resilience according to respondents' gender and marital status. **(A)** Job Enjoyment before COVID-19. **(B)** Job Enjoyment during COVID-19. **(C)** Well-being. **(D)** Resilience were compared using Welch's *t* test or Mann–Whitney U test. ***p < 0.001; **p < 0.01, *p < 0.05.

Married women had lower levels of self-reported wellbeing than married men, while there were no other gender differences between responders who were single and in partnership (Figure 1A). When looking at gender differences, married women reported lower levels of wellbeing when compared to married men (*P* = 0.028), and single females reported significantly lower levels of wellbeing than both married women and married men (*P* = 0.017 and *P* < 0.0001, respectively).

Married staff, irrespective of gender, perceived their resilience as significantly higher than staff who were single or in partnership. No differences were found in staff that are in partnerships vs. those who are single with regard to self-assessed resilience (Figure 1B). When considering the effect of gender, significant lower resilience was reported by single compared to married female staff (*P* = 0.007) or married male staff (*P* = 0.011).

Married staff perceived their job enjoyment as higher than those who were single. There were no significant differences between married staff and those who were in partnerships. No significant differences were found between those who are single and those who are in partnerships (Figure 1C). No differences were found in job enjoyment post COVID for all marital groups (Figure 1D).

### Survey Outcomes When Controlling for the Respondents' Marital Status

Regression analysis suggests that male respondents have a positive association with higher self-assessed wellbeing score compared to respondents with other genders (*p* = 0.014) disregard of their marital status. Interestingly, being female respondents have a significantly positive association (*P* = 3.35e10^−5^) higher satisfaction with time outside work before COVID-19 but this difference cannot be observed during the COVID-19. Moreover, by controlling marital status, respondents with age over 60 have a strong association with having a higher self-assessed wellbeing (*P* = 0.032) and resilience (*P* = 0.003).

### Impact of Professional Role on Survey Outcomes

When looking at differences between staff in patient versus no patient facing roles, no clear difference can be observed in terms of job enjoyment, satisfaction outside work, well-being, resilience and redeployment-related anxiety between patient facing roles and no patient facing roles.

In our survey, there were 258 (71%) respondents who continued to provide modified service in their clinical specialty or for non-COVID-19 patients during the pandemic. The professional satisfaction for the modified service of respondents taking patient facing roles was significantly lower than those with non-patient facing responsibilities (*P* = 0.019). Tele-medicine was included in the provide modified clinical service of 176/365 survey respondents. Specifically, rheumatologists providing a tele-medicine service (*n* = 38) had significantly lower professional satisfaction for the modified service than other healthcare professional providing tele-medicine (*P* = 0.007), with the caveat of a much-reduced sample size.

### Assessment of Impact of Time (May-June 2020 vs. September-October 2020) on Self-Reported Resilience and Wellbeing

**Figure 2:**
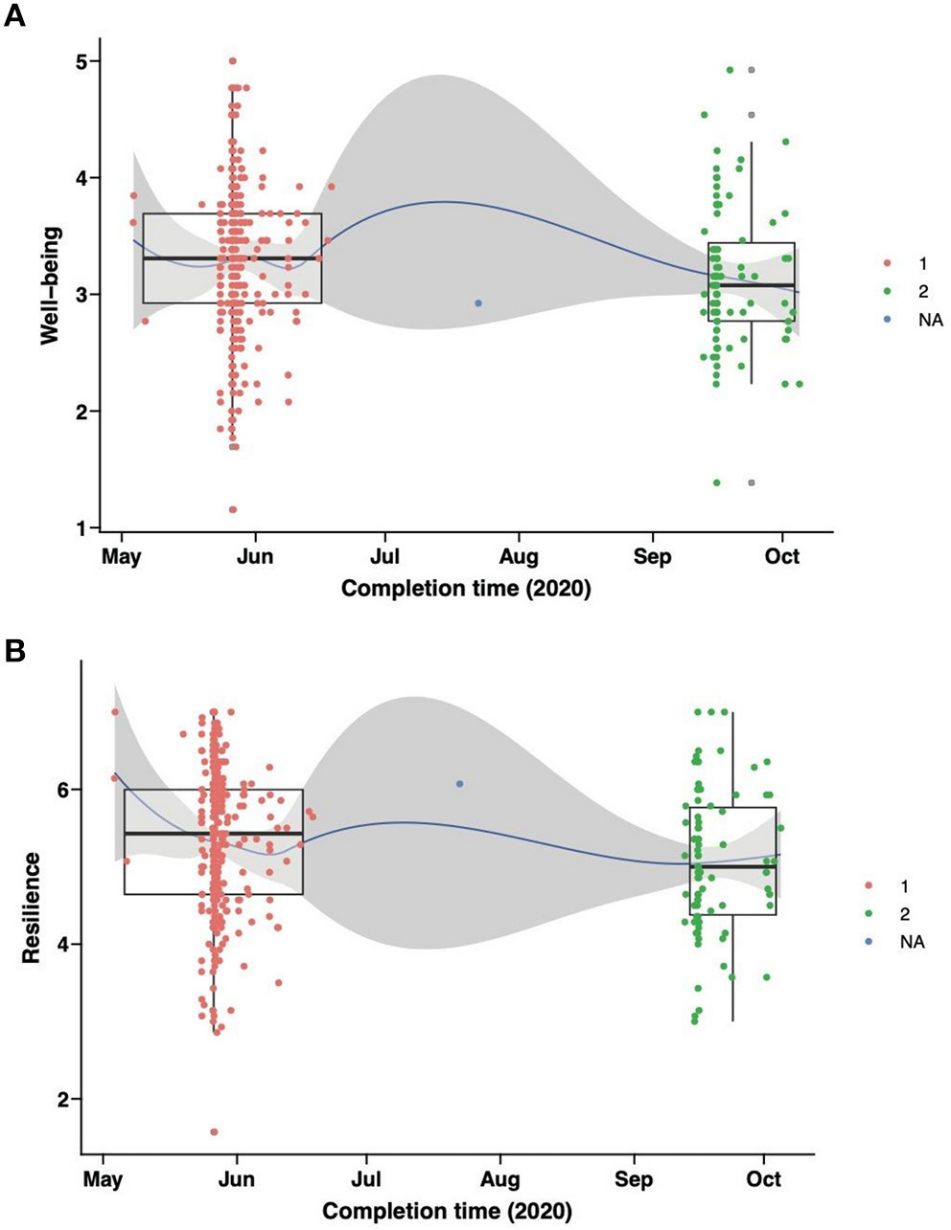
Assessment of impact of time (May-June 2020 vs. September-October 2020) on self-reported well-being and resilience. Box plots and scatter plots show comparisons of **(A)** well-being and **(B)** resilience between two groups of staffs completing questionnaires during May-June 2020 (in red) or September-October 2020 (in green). Area in grey indicates 95% confidence interval.

Self-assessed wellbeing and resilience was measured over time for all survey respondents. As expected, the majority of the responses were collected when the survey went live (May 2020) and after a reminder to complete the survey was sent out via social media in September 2020). Self-assessed wellbeing in May 2020 was found to be significantly higher than that in September 2020 (3.308 vs. 3.077, *P* = 0.045) (Figure 2A). Similar result observed with significantly higher self-assessed resilience in May than that in September 2020 (5.429 vs. 5.000, *P* = 0.014) (Figure 2B).

## Discussion

Unsurprisingly, our research participants reported fall in job enjoyment during the COVID-19 pandemic, compared to their recall of pre-pandemic job enjoyment. This was a consistent finding for all of the staff surveyed and echo similar findings in a number of international studies (41–43). One study conducted by the British Medical Association (BMA) found that 59% of doctors described their level of exhaustion from work during the pandemic as “higher than normal” in October 2020, despite the ease of the pandemic restriction (44). The participants in our survey had rated work fulfillment and recognition highly in the first wave of the COVID-19 pandemic which could explain the perceived increase in job satisfaction, whereas other publication provided evidence that doctors in the UK rated their feelings of being valued for their work during the pandemic quite low: 2.84 out of 5 (44). This disparity in perceived work recognition may be a factor influencing differences in job satisfaction globally. Other explanations for low job satisfaction in HCWs during the pandemic were perceived job inequalities (45), type of work environment (hospital vs. community) (43), as well as individual factors such as emotional intelligence (46). Interestingly, we did not find any differences between different professional roles, suggesting that the COVID-19 pandemic affected staff beyond the daily jobs. This may reflect the chronic occupational stress in university workers (47) in relation to various factors such as family-work balance and academic burnout (48, 49), accentuated by the additional psychological stress related to the teaching and academic life disruptions in the context of COVID-19 pandemic (50).

There have been limited research on the relationship between job satisfaction and marital status both during and before the pandemic. In this study, married HCWs recalled higher levels of pre-pandemic job enjoyment than single staff. However, this is clearly not a universal pattern, as a similarly designed study in Laos found no significant difference between married and single HCWs (51). Conversely, our results showed no significant difference between the job enjoyment of married staff and single staff during the pandemic, which contrasts with a study from Vietnam which found that married staff working closer to patients during the COVID-19 pandemic had a higher job satisfaction (52). These discrepancies suggest that, when the research is targeting staff support in a particular country or health care system, then comparisons between vast regions of the world may not be very meaningful, if at all. On the other hand, if the purpose of the research is to study the macro conditions affecting health care staff wellbeing, then it is useful to identify regional differences in staff experience.

The relationship between marital status and wellbeing is more consistent across the existent literature (19, 21, 22), with the general trend of lower rates of wellbeing for single HCWs. Our survey also found that married individuals had higher rates of wellbeing than those in a partnership. This could be potentially explained by the increased likelihood of married responders to live with their partner than those in a relationship, especially in the context of quarantine restrictions associated with the COVID-19 pandemic, providing them an easier access to social support. Social support has also shown to be a protective factor for mental health in HCWs during the pandemic (53). Female responders, regardless of marital status perceived their wellbeing as lower than their male counterparts during the COVID-19 pandemic (6–8, 11, 16–18, 20). Our study also provided evidence that single females self-reported lower levels of wellbeing when compared to married women and men alike, similarly to another study from Italy (19).

Married HCWs in our study also rated their resilience more highly than both single HCWs and those in partnerships. Whilst there have not been previous studies comparing the resilience of HCWs in a partnership with those who were married during the pandemic, previous studies comparing married to single HCWs generated contrasting results. A study in Spain (54) and one in Iran (55) found that married HCWs had higher scores of resilience during the COVID-19 pandemic, whereas a study in Italy (56) did not find a significant difference between single and married HCWs.

Complementary to previous studies (9, 14–18), our results have found that during the pandemic, the wellbeing scores were influenced by the age of the responders, with the younger HCWs reporting lower scores. Our results also found that the reported resilience scores increased with age– an area which has had little prior exploration. One previous study found age to be the most important factor in determining resilience during the pandemic, above having children, occupation and gender, respectively (57). It was postulated that this is likely explained by the advantage of age-related experience in providing coping skills for managing emotionally challenging incidents and this theory is supported by another study which tested age and relevant experience independently (54). They found that while experience was associated with increased scores of resilience, age when tested independently to experience, was not, and future studies should aim to explore the relationship between these two factors. Another important concept for making sense of differential experiences is loss, in terms of meaningful and valued activities and relationships that is integral to life satisfaction and support identities. As well as having had less life experiences to grow resilience and coping capacity, the COVID-19 pandemic may have brought greater losses to younger people in general and younger HCWs in particular. Another psychological variable of interest is perceived control in work and outside work.

Interestingly, one factor which led to no significant differences in job enjoyment, satisfaction outside work, wellbeing, resilience and redeployment-related anxiety, was the staff's type of role: e.g., patient vs. non-patient facing occupational role during the pandemic. While this seems counterintuitive as most of the previous research suggested that increased exposure to COVID-19 pandemic decreases psychological wellbeing (5, 7, 9, 10, 18, 27), there have been a number of studies showing non-clinical staff to have lower wellbeing scores than HCWs (17, 31, 42, 58). The authors suggested that the unbalanced degrees of preparation for and support through the pandemic, could be a possible explanation for the low wellbeing scores reported by staff not directly involved in managing the pandemic.

A large proportion of our non-patient facing participants were university staff and a previous study in the US reported that staff working in academia reported a reduction in well-being since the start of the COVID-19 pandemic, however in their study the wellbeing scores were higher than those reported by the clinical staff (25). In contrast, while our study did not find patient facing HCWs to have generally lower satisfaction, we did find that HCWs in patient facing roles had lower satisfaction for modified services such as telemedicine, and this was particularly relevant for rheumatologists. This may be due to the nature of systemic manifestations looked after during rheumatological consultations, which are difficult to manage remotely, and has also been significantly affected by the survey selection bias (the survey was led by rheumatologists who have been better represented in the sample size) Another study found that 71% of telephone consultations with rheumatologists reached the same diagnostic conclusion as a face-to-face appointment, in comparison to 97% of video call consultations (59).

Furthermore, our results bring attention to the fact that wellbeing and resilience of HCWs working in the UK decreased from May 2020 to November 2020 and previous international studies have found similar results. One global meta-analysis (60) confirmed that the pooled prevalence of anxiety in HCWs during Jan-March 2020, April-June 2020 and July-Sep 2020 increased from 30 to 48% and 60.79%, respectively and the prevalence for depression during the same time periods also escalated from 32.5 to 39.62% and 46.88%, respectively. Another study in Russia (26) found that anxiety in HCWs was higher during their second peak (Oct 2020) in comparison to their first peak (May 2020) of the pandemic. As expected, these results suggest that the increased duration of the pandemic led to poorer outcomes, however, further studies are required to appreciate if this is a long-term effect. It is unsurprising that our study found both resilience and wellbeing to decrease over time as previous research confirms a positive correlation between resilience and wellbeing scores in HCWs during the pandemic (57, 61). High resilience may serve as a protective factor against emotional distress, as one study found that when satisfaction increased, resilience also increased (57), providing insight into how HCW and other staff wellbeing can be improved during challenging periods of time. An alternative hypothesis is that resilience is mood-related, so that people may feel and report greater resilience when there is an uplift to mood, and vice versa. This suggests that it is important to measure resilience by also asking about resilient behaviors and not just perceptions.

The current study adds to the growing literature regarding the effects of the COVID-19 pandemic on the mental health of HCWs and university staff. There is currently limited information on how resilience and job satisfaction of HCWs and university staff working in in the field of healthcare and healthcare research have been affected by the COVID-19 pandemic in the UK. Previous studies have not explored some of the variables we investigated here, such as comparison between being married vs. in partnership or performed a parallel evaluation of wellbeing, resilience and job satisfaction. The strength of our survey study is in the hypotheses generated for future research which, as well as focus on work-related variables (e.g., frontline, risk perceptions), should also focus on gender and age differences as these could differentially affect people's capacity to maintain meaningful relationships and a sense of control and how they experience the gains and losses as a result of drastic changes to life. Having a more specific understanding of factors likely to influence mental health outcomes and other aspects related to job satisfaction and life satisfaction more generally will hopefully allow for more effective planning of targeted interventions to support HCWs and staff working in various other professional areas during future pandemics and other health care crises.

## Limitations

The survey was cross-sectional and did not look for changes in parameters assessed over time. It has mainly been disseminated across social medial platforms and through staff emails within the departments of researchers. Therefore, selection bias can be expected. For example, the survey is likely to have missed participants that do not use/have access to social media. There was also a likely recall bias due to the retrospective nature of part of the survey, which asked individuals to think back to how they felt prior to COVID-19 pandemic. Other limitations of this study include the reduced numbers of junior staff and participants between within the age range 18–25, and the focus on one urban geographical area, as 77.7% of respondents worked in London during the COVID-19 pandemic. The job satisfaction has not been measured simply on a VASM rather than using a composite measure likely to capture more adequately the various factors contributing to work satisfaction. We were also unable to control for many other potential confounding factors, such as living alone or not during the pandemic, irrespective of the marital status, living with/caring for children, having access to network support at home or at work, or the type of professional role (as the respondents were spread across too many roles to enable a meaningful statistical analysis). The significant research and professional fatigue affecting HCWs and university staff during the COVID-19 pandemic, prevented a longer/ more granular survey design.

## Conclusion

Our study highlights a reduction in satisfaction scores of HCWs during the pandemic, in comparison to retrospective pre-pandemic scores, which affected disproportionately single staff. Being younger, female or in a patient facing role was also associated with poorer outcomes. Furthermore, we identified that wellbeing and resilience in HCWs decreased over time during the 2020 waves of the pandemic in the UK. These results can be used to support tailored interventions for categories of staff more at risk of poorer outcomes or to predict which individuals may be at higher risk in the case of future pandemics.

## Data Availability Statement

The raw data supporting the conclusions of this article will be made available by the authors, without undue reservation.

## Ethics Statement

The studies involving human participants were reviewed and approved by UK Health Research Authority approval, reference: 20/HRA/2547. The patients/participants provided their written informed consent to participate in this study.

## Author Contributions

CC, WW, PM, and JH designed the survey. CC and WW gained the study ethical approval. WW, PM, AK, LH, JH, and CC coordinated the survey dissemination and data collection. JP performed the study analysis. JP, WW, GD, NC, PM, AK, and CC wrote the first draft of the manuscript. All authors reviewed the manuscript, provided intellectual input in the study analysis and presentation of findings, and approved the final version of the manuscript.

## Funding

This work was supported by grants from the NIHR UCLH Biomedical Research Center grant BRC772/III/EJ/101350, BRC773/III/CC/101350 and was performed within the Center for Adolescent Rheumatology Versus Arthritis at UCL UCLH and GOSH supported by grants from Versus Arthritis (21593, 22908, and 20164).

## Author Disclaimer

The views expressed are those of the authors and not necessarily those of the NHS, the NIHR or the Department of Health.

## Conflict of Interest

The authors declare that the research was conducted in the absence of any commercial or financial relationships that could be construed as a potential conflict of interest.

## Publisher's Note

All claims expressed in this article are solely those of the authors and do not necessarily represent those of their affiliated organizations, or those of the publisher, the editors and the reviewers. Any product that may be evaluated in this article, or claim that may be made by its manufacturer, is not guaranteed or endorsed by the publisher.
