# Supplementary material for: Marital Status and Gender Differences as Key Determinants of COVID-19 Impact on Wellbeing, Job Satisfaction and Resilience in Health Care Workers and Staff Working in Academia in the UK During the First Wave of the Pandemic
Source: Front Public Health. 2022 Jun 27;10:928107. doi: 10.3389/fpubh.2022.928107 (PMC9271694; doi:10.3389/fpubh.2022.928107)
Supplement: Supplementary file 1 [file Table_1.DOCX]

Warwick-Edinburgh Mental Well-being Scale (WEMWBS)

**Well-being assessment**

Below are some statements about feelings and thoughts.

Please tick the box that best describes your experience of each **over the last two weeks.**

| **STATEMENT** | **None of the time** | **Rarely** | **Some of the time** | **Often** | **All of the time** |
| --- | --- | --- | --- | --- | --- |
| 1. I’ve been feeling optimistic about the future |  |  |  |  |  |
| 2. I’ve been feeling useful |  |  |  |  |  |
| 3. I’ve been feeling relaxed |  |  |  |  |  |
| 4. I’ve been feeling interested in other people |  |  |  |  |  |
| 5. I’ve had energy to spare |  |  |  |  |  |
| 6. I’ve been dealing with problems well |  |  |  |  |  |
| 7. I’ve been thinking clearly |  |  |  |  |  |
| 8. I’ve been feeling good about myself |  |  |  |  |  |
| 9. I’ve been feeling close to other people |  |  |  |  |  |
| 10. I’ve been feeling confident |  |  |  |  |  |
| 11. I’ve been able to make up my own mind about things |  |  |  |  |  |
| 12. I’ve been feeling loved |  |  |  |  |  |
| 13. I’ve been interested in new things |  |  |  |  |  |
| 14. I’ve been feeling cheerful |  |  |  |  |  |

Reference: Tennant R, Hiller L, Fishwick R, Platt S, Joseph S, Weich S, et al. The Warwick-Edinburgh Mental Well-being Scale (WEMWBS): development and UK validation. Health Qual Life Outcomes. 2007;5:63.
