# Supplementary material for: Marital Status and Gender Differences as Key Determinants of COVID-19 Impact on Wellbeing, Job Satisfaction and Resilience in Health Care Workers and Staff Working in Academia in the UK During the First Wave of the Pandemic
Source: Front Public Health. 2022 Jun 27;10:928107. doi: 10.3389/fpubh.2022.928107 (PMC9271694; doi:10.3389/fpubh.2022.928107)
Supplement: Supplementary file 2 [file Table_2.DOCX]

14-item resilience scale (RS-14)

**Assessment of Resilience**

Please read each statement and tick the box to the right of each statement that best indicates your feelings about the statement.

| **STATEMENT** | **Strongly Strongly**  **Disagree Agree** | | | | | | |
| --- | --- | --- | --- | --- | --- | --- | --- |
|  | **1** | **2** | **3** | **4** | **5** | **6** | **7** |
| 1. I usually manage one way or another |  |  |  |  |  |  |  |
| 2. I feel proud that I have accomplished things in my life |  |  |  |  |  |  |  |
| 3. I usually take things in my stride |  |  |  |  |  |  |  |
| 4. I am friends with myself |  |  |  |  |  |  |  |
| 5. I feel that I can handle many things at a time |  |  |  |  |  |  |  |
| 6. I am determined |  |  |  |  |  |  |  |
| 7. I can get through difficult times because I have experienced difficulty before |  |  |  |  |  |  |  |
| 8. I have self-discipline |  |  |  |  |  |  |  |
| 9. I keep interested in things |  |  |  |  |  |  |  |
| 10. I can usually find something to laugh about |  |  |  |  |  |  |  |
| 11. My belief in myself gets me through hard times |  |  |  |  |  |  |  |
| 12. In an emergency, I am someone people can generally rely on |  |  |  |  |  |  |  |
| 13. My life has meaning |  |  |  |  |  |  |  |
| 14. When I’m in a difficult situation, I can usually find my way out of it |  |  |  |  |  |  |  |

**Reference:** Damasio BF, Borsa JC, da Silva JP. 14-item resilience scale (RS-14): psychometric properties of the Brazilian version. J Nurs Meas. 2011;19(3):131-45.
